# Responsiveness of the Spanish Version of the “Skin Cancer Index”

**DOI:** 10.1155/2016/8180348

**Published:** 2016-10-09

**Authors:** M. de Troya-Martín, F. Rivas-Ruiz, N. Blázquez-Sánchez, I. Fernández-Canedo, M. Aguilar-Bernier, J. B. Repiso-Jiménez, J. C. Toribio-Montero, M. Jones-Caballero, J. Rhee

**Affiliations:** ^1^Department of Dermatology, Agencia Sanitaria Costa del Sol, Marbella, Spain; ^2^Red de Investigación en Servicios de Salud en Enfermedades Crónicas (REDISSEC), Spain; ^3^Unit of Investigation, Agencia Sanitaria Costa del Sol, Marbella, Spain; ^4^Department of Dermatology, University of Sydney, Sydney, NSW, Australia; ^5^Department of Otolaryngology and Communication, Sciences Medical College of Wisconsin, Milwaukee, WI, USA

## Abstract

*Background.* Skin Cancer Index (SCI) is a specific questionnaire measuring health related quality of life (HRQL) in patients with cervicofacial non-melanoma skin cancer (CFNMSC). The original scale has recently been adapted and validated into Spanish.* Objectives.* Evaluate the responsiveness of the Spanish version of SCI.* Methods.* Patients with CFNMSC candidate for surgical treatment were administered the questionnaire at time of diagnostic (*t*_0_), 7 days after surgery (*t*_1_), and 5 months after surgery (*t*_2_). The scale and subscales scores (C1: social/appearance, C2: emotional) were then evaluated. Differences between *t*_0_-*t*_1_, *t*_1_-*t*_2_, and *t*_0_-*t*_2_ were determined and a gender-and-age segmented analysis was performed.* Results.* 88 patients, 54.8% male, mean age 62.5 years, completed the study. Differences between *t*_0_-*t*_1_ and *t*_1_-*t*_2_ scores were statistically significant (*p* < 0.05). The lowest values were found at time of diagnosis and postsurgery. Women and patients under 65 years showed the lowest values at the three times.* Limitations.* Concrete geographic and cultural area. Clinical and histological variables are not analysed.* Conclusions.* Our results confirm responsiveness of the Spanish version of the SCI. Further development of the instrument in Spanish-speaking countries and populations will make it possible to extend worldwide research and knowledge horizons on skin cancer.

## 1. Introduction

Non-melanoma skin cancer (NMSC), basal cell carcinoma (BCC), and squamous cell carcinoma (SCC) are the most common malignant tumours among humans [1, 2]. Their incidence has increased dramatically over the past 20 years, especially among women and people aged 30–39 years [3, 4], as a result of excessive exposure to ultraviolet radiation [5]. Although NMSC has a low mortality rate (0.1–0.3%), its morbidity is high; in over 80% of cases it is located in the face, where the tumour itself or surgical treatment often causes functional and aesthetic problems of diverse types [6, 7]. In addition, there is an accumulated risk of around 40% of developing a second NMSC within three years [8], making this tumour a chronic and mutilating disease [9]. Health related quality of life (HRQL) is a measure of particular interest with respect to cervicofacial NMSC (CFNMSC). However, the lack of specific instruments and the low sensitivity of the questionnaires previously used have hampered understanding of this essential aspect of the disease, producing results that are sometimes confusing [10–21].

In 2005, Rhee et al. created the first specific HRQL questionnaire for patients with CFNMSC, termed the Skin Cancer Index (SCI), consisting of 15 items exploring three dimensions about HRQL in this patients (emotional, social, and appearance) [22]. In further studies the instrument demonstrated excellent psychometric properties (validity, reliability, and responsiveness) [23, 24]. The Spanish version of SCI has recently been developed, showing also an excellent level of internal consistency and an adequate level of reliability [25]. The aim of the present study is to assess responsiveness of the Spanish scale.

## 2. Material and Methods

A prospectively longitudinal study was designed and approved by the Bioethics Committee of our hospital.

Patients were selected consecutively among subjects diagnosed with CFNMSC candidate for surgical treatment at the Dermatology Service of the Costa del Sol Hospital during the period April 2009 to November 2011. All patients included in the study were new-onset patients with CFCCNM confirmed by biopsy (BCC or SCC), aged over 18 years, that correctly understand spoken and written Spanish, and who gave their informed consent to participate. Those who presented intellectual impairment or suffered a severe physical or mental illness were excluded.

The participants were invited to complete the quality-of-life questionnaire at three different time points during the surgical process: *t*_0_ (time of diagnostic confirmation), *t*_1_ (7 days after surgery), and *t*_2_ (5 months after surgery). The Spanish version of the SCI [25] is linguistically and semantically equivalent to the original scale but differs in the number of items, because during the validation process three items were dismissed by not meeting criteria of validity. The final version is composed of 12 items, with two underlying dimensions which we termed (following the original model) “social/appearance” (7 items) and “emotional” (5 items). Both components presented an excellent level of internal consistency, with Cronbach's alpha values above 0.85. In addition, the Spanish scale provided an adequate level of reliability, with weighted kappa values greater than 0.4 and percentages of absolute agreement exceeding 60% in most items. As the original version, the answers are given on a Likert 5-point scale. The standardised final score ranges from 0 (lowest quality of life) to 100 (highest quality of life).

Responsiveness was assessed as the difference in the mean score in the scale at stages *t*_0_, *t*_1_, and *t*_2_. Second outcome measure might be described as differences in the mean score in age (> or <65 y-o) and sex (male, female) groups.

### 2.1. Statistical Analysis

The global scores on the scale and its components were obtained at each of the three time points. To assess the sensitivity to change of the instrument, the paired Student's* t*-test was used (or the Mann-Whitney test if the criteria for parametric testing were not met). We recorded the differences of the means (DM) of the scores for the scale and its components and the corresponding 95% confidence intervals (95% CI). In addition, an age-and-gender segmented analysis was performed. The level of significance was set at *p* < 0.05. The statistical analysis was carried out using SPSS v15.

## 3. Results

88 of the 100 patients included in the study completed the survey at all three time points. Of these respondents, 54.8% were men, with a mean age of 62.5 years (SD: 14.1). On a standardized scale 0–100, the mean total scores were 59.2 (*t*_0_), 63.9 (*t*_1_), and 75.3 (*t*_2_). The mean scores for the CI scale component were 79.4 (*t*_0_), 84.4 (*t*_1_), and 90.5 (*t*_2_), and those for the C2 component were 31.0 (*t*_0_), 35.1 (*t*_1_), and 54.1 (*t*_2_) (Table 1).

The differences in the total score for the scale, in all the pairwise comparisons, were statistically significant: *t*_0_ versus *t*_1_ (DM: 2.24; 95% CI 0.63–3.84), *t*_0_ versus *t*_2_ (DM: 7.73; 95% CI 5.80–9.66), and *t*_1_ versus *t*_2_ (DM: 5.49; 95% CI 3.86–7.11), except for *t*_0_-*t*_1_ in C2 (Table 2).

In the gender-and age segmented analysis, women and subjects younger than 65 years had lower scores at all three time points, and the changes over time were statistically significant in all tests for *t*_0_-*t*_2_ and *t*_1_-*t*_2_, except for *t*_1_-*t*_2_ in C1 in the subjects aged over 65 years (Tables 3 and 4).

## 4. Discussion

Responsiveness, defined as “the ability of an instrument to detect change over time in the construct to be measured,” is the third main psychometric property, together with validity and reliability, to consider in health related questionnaires [26, 27].

Our results confirm responsiveness of the Spanish version of the SCI. We have used the same methodology the authors did to test responsiveness of the original questionnaire [16], following the recommendations of the international guidelines for validating health questionnaires [26–28]. Our study has been carried out in patients with CFNMSC undergoing surgery at three different time points of the medical care process. For the overall scale and the subscales, the capacity of the instrument to identify changes in the subjects' HRQL has been revealed.

Like similar studies in the USA, the UK, or Canada [16, 29–31], patients with CFNMSC experienced a significant impact on their HRQL at the moment of diagnosis, and surgical treatment produces a marked improvement, as indicated by the significant increase in the scale score. HRQL was found to be more severely affected among female patients and patients of both sexes aged under 65 years, as reported by Rhee et al. [16]. Unlike other studies conducted in Anglo-Saxon countries [16, 29, 30], the values for emotional subscale were considerably lower than those for the social-appearance component, for all time points and all groups of patients.

As this is the only version of the scale measuring HRQL in patients with NMSC developed in a language other than the original, its implementation in countries and populations belonging to a Spanish-language culture will make it possible to extend worldwide research horizons of the disease.

This is a single-center study conducted in a particular sociocultural context. Therefore, our data need to be confirmed, by extending this investigation to other areas of Spain and to Latin American countries.

In conclusion, our results confirm the ability of the Spanish version of the SCI to discriminate changes in the HRQL of patients with CFNMSC. In the future, its implementation in Spanish-speaking countries and populations will make it possible to extend worldwide research on skin cancer.

## Figures and Tables

**Table 1:** Scale results for all patients assessed (*n* = 88).

	*t*_0_		*t*_1_		*t*_2_	
	Mean	SD	Mean	SD	Mean	SD
Total	59.2	21.2	63.9	20.2	75.3	20.2
C1	79.4	24.8	84.4	23.4	90.5	19.5
C2	31.0	27.0	35.1	28.0	54.1	31.5

**Table 2:** Sensitivity to change.

	Mean	95% confidence interval		*p*
		Lower	Upper	
*Total for the Scale*				
Difference *t*_0_-*t*_1_	4.66	1.32	8.01	*0.007*
Difference *t*_0_-*t*_2_	16.10	12.08	20.12	*<0.001*
Difference *t*_1_-*t*_2_	11.43	8.05	14.81	*<0.001*

*Component 1*				
Difference *t*_0_-*t*_1_	3.98	0.78	7.18	*0.015*
Difference *t*_0_-*t*_2_	8.86	5.47	12.26	*<0.001*
Difference *t*_1_-*t*_2_	4.84	2.41	7.26	*<0.001*

*Component 2*				
Difference *t*_0_-*t*_1_	3.42	−0.73	7.56	*0.105*
Difference *t*_0_-*t*_2_	18.50	13.29	23.71	*<0.001*
Difference *t*_1_-*t*_2_	15.18	10.11	20.25	*<0.001*

**Table 3:** Scale results, segmented by sex (*n* = 88).

	*t*_0_		*t*_1_		*t*_2_		*p* *t*_0_-*t*_1_	*p* *t*_0_-*t*_2_	*p* *t*_1_-*t*_2_
	Mean	SD	Mean	SD	Mean	SD			
Total									
Male	61.6	22.5	65.2	20.8	78.9	20.5	*0.073*	<0.001	<0.001
Female	57.4	19.1	62.9	19.3	70.6	20.0	*0.081*	<0.001	*0.005*

C1									
Male	82.8	24.0	86.8	22.2	92.3	18.2	*0.021*	<0.001	*0.007*
Female	76.3	25.1	82.5	24.9	88.3	21.8	*0.15*	*0.005*	*0.029*

C2									
Male	32.1	30.2	35.0	30.5	60.1	32.4	*0.425*	<0.001	<0.001
Female	30.9	23.7	35.5	25.2	45.8	29.7	*0.278*	*0.002*	*0.014*

**Table 4:** Scale results, segmented by age (*n* = 88).

	*t*_0_		*t*_1_		*t*_2_		*p* *t*_0_-*t*_1_	*p* *t*_0_-*t*_2_	*p* *t*_1_-*t*_2_
	Mean	SD	Mean	SD	Mean	SD			
Total									
<65	55.0	20.0	58.7	19.5	70.7	20.0	*0.166*	<0.001	<0.001
≥65	62.6	21.6	68.3	19.8	79.3	19.7	*0.014*	<0.001	<0.001

C1									
<65	75.8	26.2	79.2	24.7	88.0	20.8	*0.293*	0.001	*0.001*
≥65	82.4	23.3	89.2	21.3	92.6	18.3	*0.015*	<0.001	*0.08*

C2									
<65	25.9	21.5	30.1	25.5	46.5	30.8	*0.241*	<0.001	0.001
≥65	35.0	30.7	39.1	29.5	60.7	30.7	*0.316*	<0.001	<0.001

## Abbreviations

NMSC: Non-melanoma skin cancer
BCC: Basal cell carcinoma
SCC: Squamous cell carcinoma
CFNMSC: Cervicofacial non-melanoma skin cancer
SCI: Skin Cancer Index
HRQL: Health related quality of life.
